# Genomic Characterization of a Mercury Resistant *Arthrobacter* sp. H-02-3 Reveals the Presence of Heavy Metal and Antibiotic Resistance Determinants

**DOI:** 10.3389/fmicb.2019.03039

**Published:** 2020-01-17

**Authors:** Ashish Pathak, Rajneesh Jaswal, Ashvini Chauhan

**Affiliations:** Environmental Biotechnology Laboratory, School of the Environment, FSH Science Research Center, Florida A&M University, Tallahassee, FL, United States

**Keywords:** mercury, whole genome sequencing, comparative genomics, *Arthrobacter*, resistome

## Abstract

Nuclear production and industrial activities led to widespread contamination of the Department of Energy (DOE) managed Savannah River Site (SRS), located in South Carolina, United States. The H-02 wetland system was constructed in 2007 for the treatment of industrial and storm water runoff from the SRS Tritium Facility. Albeit at low levels, mercury (Hg) has been detected in the soils of the H-02 wetland ecosystem. In anoxic sediments, Hg is typically methylated by anaerobic microbiota, forming the highly neurotoxic methylmercury (MeHg), which biomagnifies across food webs. However, in surficial oxic wetland soils, microbially mediated demethylation and/or volatilization processes can transform Hg^2+^ into the less toxic Hg^0^ form which is released into the atmosphere, thus circumventing MeHg formation. To obtain a deeper understanding on bacterial Hg volatilization, a robust Hg-resistant (HgR) bacteria, called as strain H-02-3 was isolated from the H-02 soils. A draft genome sequence of this strain was obtained at a coverage of 700×, which assembled in 44 contigs with an N50 of 171,569 bp. The genomic size of the strain H-02-3 was 4,708,612 bp with a total number of 4,240 genes; phylogenomic analysis revealed the strain as an Arthrobacter species. Comparative genomics revealed the presence of 1100 unique genes in strain H-02-3, representing 26.7% of the total genome; many identified previously as metal resistance genes (MRGs). Specific to Hg-cycling, the presence of mercuric ion reductase (*merA*), the organomercurial lyase (*merB*), and the mercuric resistance operon regulatory protein, were identified. By inference, it can be proposed that the organomercurial lyase facilitates the demethylation of MeHg into Hg^2+^ which is then reduced to Hg^0^ by MerA in strain H-02-3. Furthermore, gene prediction using resistome analysis of strain H-02-3 revealed the presence of several antibiotic resistance genes (ARGs), that statistically correlated with the presence of metal resistant genes (MRGs), suggesting co-occurrence patterns of MRGs and ARGs in the strain. Overall, this study delineates environmentally beneficial traits that likely facilitates survival of *Arthrobacter* sp. H-02-3 within the H-02 wetland soil. Finally, this study also highlights the largely ignored public health risk associated with the co-development of ARGs and MRGs in bacteria native to historically contaminated soils.

## Introduction

Savannah River Site (SRS) managed by the United States Department of Energy (DoE), located in South Carolina (SC), United States, is a former nuclear legacy site. Mercury (Hg) contamination occurred at this site from previous industrial and nuclear production activities, and the contamination is still pervasive. Specifically, the SRS was heavily contaminated by Hg-containing discharges due to the industrial activities from 1960s to 1980s (Smith, 2007; Kuhne, 2015) as well as from a chloralkali facility near Augusta (GA, United States) that released approximately 18,000 lbs. of Hg into the Savannah River (Smith, 2007; Kuhne, 2015). Consequently, Hg contamination continues to pose serious threats to ecosystem and public health in these environments. Not only is Hg toxic as a heavy metal but it can also be microbially methylated to the neurotoxic methylmercury (MeHg) form, which is bioaccumulative and biomagnifies across aquatic food webs.

To remediate metal contaminants and improve water quality at the SRS, biogeochemical processes within the constructed wetlands have been shown to be an effective strategy (Xu and Mills, 2018). Soil samples for this study originated from the H-02 site, which is a constructed wetland (Mills et al., 2011), adjacent to the SRS H-Area, which is part of the Hazardous Waste Management Facility Operable Unit (HWMF-OU), located in the central portion of SRS. Studies conducted on the operation of the H-02 constructed wetlands have demonstrated sequestration of heavy metals (eg., copper, lead, and zinc), which are removed by adsorption to organic matter and clay particles, followed by sulfate reducing bacteria facilitating the precipitation of metal ions in the anaerobic soils (Nelson et al., 2006; Mills et al., 2011). However, recent soil measurements at the H-02 site have revealed the presence of both, Hg and MeHg, albeit at low levels (Xu and Mills, 2018). One mechanism for the removal of MeHg is biodegradation by aerobic, soil-borne microbiota that possess the required gene determinant(s). In this context, bacteria that resist high levels of MeHg are known to code the following two enzymes within their *mer* operon performing reductive demethylation of mercury- MerA and MerB (Barkay et al., 2003). The *mer* operon can be plasmid(s)-borne, or located on the chromosome(s), and can even be components of mobile genetic elements such as integrons or transposons (Summers and Silver, 1972; Hamlett et al., 1992; Liebert et al., 1999; Barkay et al., 2003).

Depending upon the presence of these *mer* gene determinants, HgR has been classified into two categories: broad spectrum and narrow spectrum. The broad spectrum HgR bacteria carry the *merB* gene, which codes for an organomercurial lyase, and demethylates MeHg into Hg^2+^, which is further reduced to Hg^0^ by the action of MerA (Barkay et al., 2003). The narrow spectrum mercury resistant bacteria do not carry the *merB* gene but only harbors the *merA* gene. Thus, MerA performs a central role in different types of Hg resistant (HgR) bacteria in the environment. Further, many *mer* operons also comprise of genes for the transcriptional regulators MerR and MerD, along with *merT*, *merC*, and *merF* which encode for Hg transporters, as well as additional Hg transfer proteins encoded by *merP* and *merE* (Barkay et al., 2003; Boyd and Barkay, 2012). Given the bioremediative properties of these Hg-demethylating enzymes, there is considerable interest in genome-enabled assessment of aerobic mercury-resistant bacteria (HgR bacteria).

As part of an ongoing study on heavy metal-microbe interactions, we have demonstrated that long-term exposure of co-contaminants present in the impacted SRS ecosystems to naturally occurring environmental microbiota has enabled acquisition of an arsenal of metal resistant genes (MRGs), that can also be referred to asas the metal pollutome (Spînu et al., 2017), thus conferring tolerance and/or resistance against heavy metals such as uranium (U) and Hg (Benyehuda et al., 2003). Furthermore, a growing body of research, including our recent work (Agarwal et al., 2019), has demonstrated the propensity of metal contamination to be a facilitator for the recruitment of antibiotic resistance genes (ARGs) or the “resistome” within the native soil microbiota (Ji et al., 2012; Seiler and Berendonk, 2012; Zhu et al., 2013), leading to the rapid emergence and perseverance of multidrug-resistant (MDR) bacteria, commonly referred to as “superbugs” (Davies and Davies, 2010). Toward this end, a team commissioned by the United Kingdom government projected that antimicrobial resistance (AMR) could be the leading cause of approximately 10 million deaths a year by the year 2050 (O’Neill, 2014), outpacing cancer, which is the current largest cause of human mortality.

The environmental nexus between MRGs and the antibiotic resistant genes (a.k.a the resistomes or ARGs) are now being widely studied especially in contaminated soils (Baker-Austin et al., 2006; Knapp et al., 2011; Ji et al., 2012; Seiler and Berendonk, 2012). This area of research is particularly relevant to DOE sites contaminated with heavy metals, which we believe are likely serving as an evolutionary repository for proliferation of multiple antibiotic resistant microbes, which presents a major public health concern. The MRGs and ARGs are typically co-selected in the environment through the several mechanisms; (1) co-resistance- owing to close location of the resistome encoding for antibiotics and/or metal resistance, e.g., are present on the same genetic element such as a plasmid or are present in the same cell (e.g., *merA* and *KPC* beta-lactamase; (2) cross-resistance- when resistance functions of efflux as well as antibiotic resistance are offered by a single resistome (e.g., *mdrL* confers resistance to metals, such as zinc, cobalt, and chromium along with antibiotics, such as erythromycin, josamycin, and clindamycin (Chapman, 2003); and, (3) co-regulatory resistance- when a single regulatory gene element controls multiple resistance gene determinants conferring resistance to different toxic compounds, including antibiotics, biocides, and metals (e.g., *czcR* regulating expression of *CzcCBA* efflux pump, resulting in the resistance to zinc, cadmium, and cobalt along with co-regulating resistance to carbapenem antibiotics) (Perron et al., 2004). Therefore, it is critical to obtain a deeper understanding into the genome-enabled mechanisms that facilitate survival against heavy metals and antimicrobials in a historically contaminated soil environment, such as the DOE managed SRS ecosystem.

To procure HgR bacteria, to be used as model systems to understand aerobic cycling of Hg and evaluate resistomes in the SRS contaminated ecosystems, soil samples were collected from an SRS constructed wetland site called H-02. Using serial soil dilutions onto media containing Hg chloride, we obtained several bacterial strains, one of which was identified as *Arthrobacter* sp. H-02-3. Notably, our ongoing research previously revealed the predominance of *Burkholderia* spp. and *Arthrobacter* spp. in SRS contaminated soils (Jaswal et al., 2019). This is mainly due to the fact that the soil-borne actinomycetes in the *Arthrobacter* genus are known for their metabolic ability to resist heavy metals including U and Ni (Tsuruta, 2007; Margesin and Schinner, 1996) and degrade a variety of xenobiotic compounds (O’Loughlin et al., 1999; Marx and Aitken, 2000; Juhas et al., 2009; Chen et al., 2016; Kulkarni et al., 2016). In fact, another *Arthrobacter* strain isolated in our lab from the SRS soils, SRS-W-1-2016, was found to contain a plethora of gene determinants including several gene homologs such as efflux pumps and MFS transporters, known to provide resistance against heavy metal/radionuclides, P-type ATPase translocators and heavy metal-responsive transcriptional regulators (Chauhan et al., 2018). In addition, bacterial resistance to metals is also regulated by genes for the RND family proteins, which provide for resistance, nodulation, and cell division, respectively. All these proteins are a part of transenvelope protein complex whose major function is to detoxify the cellular environment by transporting the periplasmic toxic metal cations to the outside of the cell. In our previous study several RND-type efflux gene homologs have been reported to be interspersed within the genome of *Arthrobacter* strain SRS-W-1-2016, to maintain metal homeostasis and survival in the heavily co-contaminated SRS soil habitat. Our overarching objective is to develop a deeper understanding on the microbially mediated bioremediation of heavy metals, which is of significant interest for better stewardship of the SRS ecosystem. Due to the discharge of Hg-laden wastes by the Chloralkali plants and other industrial processes located near SRS (Kvartek et al., 1994), into the surrounding water bodies, Hg still poses a significant public health risk. Mixed-contaminated environments, such as SRS, can be bioremediated using HgR bacteria (Figueiredo et al., 2014) and fungi (Hindersah et al., 2018), resulting in wastewaters suitable for discharge directly into the environment (Wagner-Döbler, 2003).

Therefore, the objectives of this study were as follows: (1) to isolate aerobic HgR bacteria from the H-02 wetland soil habitat and obtain a genome-wide understanding in context to Hg cycling; (2) provide insights into the taxonomic and evolutionary traits of the isolated HgR bacteria, native to the constructed wetland system and, (3) evaluate co-occurring MRG and ARG patterns within the isolated HgR microbiota. Overall, we propose that the identified gene determinants and evolutionary traits identified in the newly isolated *Arthrobacter* sp. H-02-3 likely enable successful colonization and potential demethylation of Hg within the contaminated SRS soil niches. Finally, this study also highlights the largely ignored public health risk associated with the co-occurrence of ARGs and MRGs in bacteria native to the historically contaminated SRS soil environment.

## Experimental Section

### Isolation and Physiological Growth Studies of Strain H-02-3

The H-02 wetland system at SRS was constructed in 2006 in order to treat the process water released from the Tritium Processing Facility, as well as stormwater runoff. The H-02 wetland system has been demonstrated to also remove heavy metals, specifically Cu, and Zn, before the water enters the Upper Three Runs Creek, a regulated stream that merges into the Savannah River. One 48 mm diameter core containing at 5–10 cm of surficial sediment below the flocculent layer was collected from the middle section in each wetland treatment cells of the H-02 constructed wetland in December 2017.

In our recent study, we collected Hg contaminated soil samples from the H-02 wetland area and isolated bacterial strains resistant to high concentrations of Hg, including *Arthrobacter* sp. H-02-3 (Pathak et al., 2017). Briefly, Hg (5 μg/ml) supplemented LB agar was used to plate out serially diluted slurry from the contaminated soil samples. A nutrient-rich media (LB) was selected over mineral salts media to isolate Hg resistant microorganism to allow for the growth of diverse groups of bacteria which is usually limited by minimal media, which may only select a narrow range of soil microbiota.

Hg resistance of the strain was evaluated by growing the bacteria on variable Hg concentrations using the Bioscreen C system (Growth Curves United States, Piscataway, NJ, United States). Variable concentrations of Hg (ranging from 0 to 50 μg/ml) were added to 4M media (Van Nostrand et al., 2007), modified with the addition of 0.04% yeast extract. The assay was performed using the 300 well honeycomb Bioscreen C plates containing 290 μl modified 4M medium and 10 μl of the inoculum (at OD_600_ of 0.45–0.5), to make a total starting volume of 100 μl in each well. The plate was incubated in the Bioscreen C instrument at 28°C under regular shaking, and OD_600_ was automatically recorded every 4 h for 4 days. This experiment was performed in triplicates and average values are reported.

### Nucleotide Sequence Accession Number

The draft genome sequence of *Arthrobacter* sp. strain H-02-3 reported in this study has been deposited at DDBJ/ENA/GenBank under the accession PYUI00000000 (https://www.ncbi.nlm.nih.gov/nuccore/PYUI00000000); Bio Project: PRJNA445730 (https://www.ncbi.nlm.nih.gov/bioproject/PRJNA445730); BioSample: SAMN08797595 (https://www.ncbi. nlm.nih.gov/biosample/8797595). This WGS version of the project consists of sequences PYUI01000001-PYUI01000044.

### Genomic Characterization and Comparative Genomics of Strain H-02-3

To understand the genomic characteristics of strain H-02-3 in reference to Hg cycling, a single colony of the bacterium growing on LB + Hg plate was inoculated into liquid LB media and incubated overnight at 30°C in a shaker at 100 rpm. After overnight growth, the media was centrifuged at 7500 rpm for 10 min to obtain a pellet, which was used for DNA extraction with Qiagen’s DNeasy PowerLyzer Kit and sequenced using an Illumina HiSeq2000 instrument (Chauhan et al., 2018). *De novo* assembly of the raw reads was performed with the SPAdes assembler, at default settings (Bankevich et al., 2012). SPAdes assembly is based on the following four stages: stage 1 (assembly graph construction) utilizing a new bulge/tip removal algorithm, as well as detection and removal of chimeric reads, aggregating biread information into distance histograms; stage 2 (k-bimer adjustment) which results in the derivation of accurate distance estimates between k-mers in the genome; stage 3 in which construction of the paired assembly graph takes place and; stage 4 in which contig construction takes place. Bowtie2 was then used to compute assembly coverage statistics by mapping raw reads to the assembled genome (Langmead and Salzberg, 2012). Specifically, a coverage filter was determined for the contigs derived from the distribution of coverage levels across the assembly. The contigs were arranged in order according to the coverage, and cumulative assembly length was computed across all contigs. Then the coverage level was determined at 50% of the total cumulative assembly length; half of which was selected as a coverage filter. The closest taxonomic relative by BLAST of the 16S rRNA sequence identified *Arthrobacter* sp. Hiyo8 DNA (accession number AP014719.1), which was used to align the remaining reads with NUCmer (Bankevich et al., 2012). Mummer plot was used to align all the contigs to these references, and the optimal contig ordering and orientation to most closely match the reference with specified layout (Bankevich et al., 2012). Contigs were then reordered and reversed as needed to match the ordering determined by mummer plot.

CGView Comparison Tool was used to generate the circular genomic map of strain H-02-3 (Grant et al., 2012). The genome, with a 700× coverage was annotated and the genes were predicted by Rapid Annotations using Subsystems Technology-RAST (Aziz et al., 2008) and NCBI’s Prokaryotic Genomes Automatic Annotation Pipeline (PGAAP), version 2.0. Gene predictions and comparisons of strain H-02-3 with other sequenced *Arthrobacter* spp., were also performed by tools offered by NCBI (Tatusova et al., 2016)^1^ and RAST, using default settings. Venn diagram and phylogenomic comparisons were obtained using the EDGAR pipeline (Blom et al., 2016). After EDGAR analysis, the Newick phylogenomic tree file was downloaded and a nucleotide based tree was constructed using MEGAX (Kumar et al., 2018; Wattam et al., 2017). Island Viewer^2^ was used to locate chromosomal deviations in GC content, also known as genomic islands (GEIs) (Yung et al., 2015). From the whole genome sequence of strain H-02-3, *merAB* genes were manually queried for homology against other bacteria using BacMet, a repository of antibacterial biocide-resistance and metal-resistance genes (Pal et al., 2014). BacMet predicted database was used for this BLASTp based analysis.

For the phylogenetic evaluation of the MerA gene in strain H-02-3, PATRIC was used with default settings (Wattam et al., 2017). After PATRIC analysis, Newick file was downloaded and a nucleotide based phylogenetic tree was constructed using default settings in MEGAX (Wattam et al., 2017; Kumar et al., 2018). Phage remnants within the whole genome sequence of strain H-02-3 were querried using the PHAge Search Tool Enhanced Release (PHASTER) pipeline (Arndt et al., 2016). Default settings were used to run this analysis.

### Statistical Analysis of Strain H-02-3

The number of genes annotated by PATRIC for HgR, AMR, efflux, and universal stress response proteins in strain H-02-3 were querried from the whole genome sequence relative to three closest phylogenetic relatives. The relatives were chosen based on the *merA* gene homology and analyzed statistically using PRIMER6 (PRIMER-E Ltd). Statistical relationships were evaluated and confirmed by principal coordinate analysis (PCoA), with the Bray-Curtis similarity index to evaluate correlations, especially between the different ARGs and MRGs between strain H-02-3 and the selected phylogenetic relatives.

## Results and Discussion

### Isolation of Strain H-02-3

Soils from a constructed wetland located in the SRS were serially diluted soils and spread plated onto LB agar plates supplemented with a concentration of 5 μg/ml Hg. Plates were incubated at 30°C, for 48 h and colonies that appeared on the plates were further purified. A vigorously growing HgR strain was obtained and labeled as strain H-02-3.

### Mercury Resistance Potential of Strain H-02-3

**FIGURE 1:**
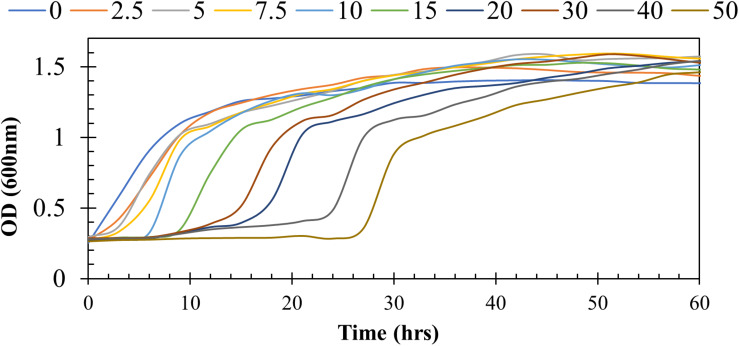
Shown are the growth responses of *Arthrobacter* sp. strain HO-2-3 at mercury (Hg) supplemented at concentrations of 0 to 50 μg/ml. Resistance was marked by growth measured at OD_600_ over time.

To determine the resistance potential of strain H-02-3 against Hg, the bacteria was grown in modified 4M media supplemented with different concentrations of Hg and the results are presented in Figure 1. It was observed that strain H-02-3 could resist up to 50 μg/ml Hg, which revealed the strong HgR ability of strain H-02-3, therefore further in-depth studies on this strain were performed.

### Genome-Centric Evaluation of Strain H-02-3

**FIGURE 2:**
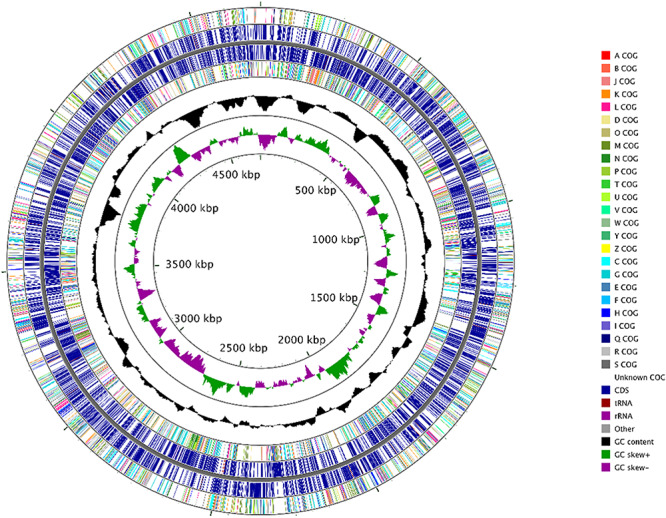
Circular genomic map of *Arthrobacter* sp. strain H-02-3 with the first (outermost) and fourth rings depicting COG categories of protein coding genes on the forward and reverse strands, respectively. The second and third rings show the locations of protein coding, tRNA, and rRNA genes on the forward and reverse strands, respectively. The black plot depicts GC content with the peaks extending toward the outside of the circle representing GC content above the genome average, whereas those extending toward the center mark segments with GC content lower than the genome average. The innermost plot depicts GC skew. Both base composition plots were generated using a sliding window of 50,000 nt.

Genomic sequences of strain H02-3, with a coverage of 700× were obtained, and characterized by the following parameters: contig count (44); total length (47,08,612 bp); longest read (5,06,972 bp); N50 (171,569 bp); N75 (95,110 bp) and N90 (62,502 bp), respectively. RAST annotation revealed the genomic size of strain H-02-3 was estimated to be approximately 4.7 Mb with 4,240 genes having a G + C content of 66.8%. Moreover, the strain comprised of a total 4,252 coding sequences, including 51 tRNA genes and two copies of the 16S rRNA genes, respectively. Figure 2 shows a circular genomic map of strain H-02-3 constructed with CGView Comparison Tool, using the 44 contigs with N_50_ contig length of 171,569 bp. Genome-centric evaluation of H-02-3 revealed that out of 4,318 total protein coding genes, 64.75% genes were associated with COGs (clusters of orthologous groups of protein); 74.34% of annotated genes were protein coding function predictions; 24.36% genes were annotated as protein coding genes with enzyme production and 25.59% genes were associated with KEGG pathways. It must be noted that COGs represent an ortholog or direct evolutionary counterpart among bacterial genomes as they evolve over time.

PATRIC-based annotation revealed the presence of 1,518 hypothetical proteins and 2,861 proteins with functional assignments; which included 1,037 proteins with Enzyme Commission (EC) numbers, 900 with Gene Ontology (GO) assignments, and 810 proteins mapped to KEGG pathways. PATRIC annotation includes two types of protein families, and the genome of strain H-02-3 possessed 4,313 proteins each belonging to the genus-specific protein families (PLFams) and the cross-genus protein families (PGFams), respectively.

**FIGURE 3:**
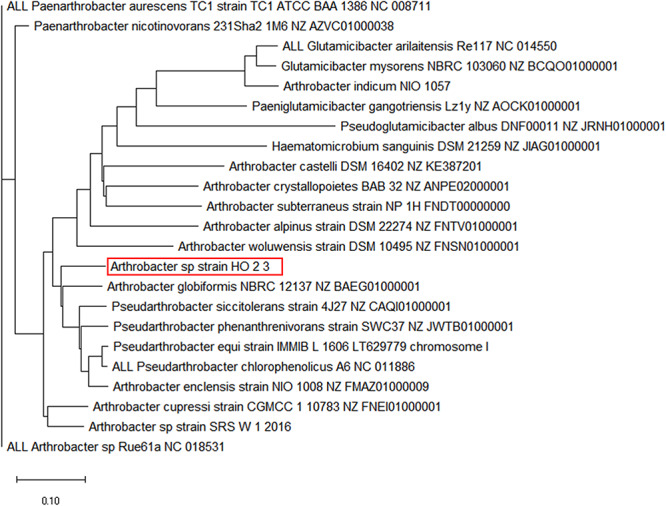
A genome-wide phylogenomic tree generated using the EDGAR pipeline workflow is shown depicting the closest taxonomic relatives of *Arthrobacter* sp. strain H-02-3. The EDGAR pipeline analyzes phylogenetic relationships between genomes based on the presence of orthologous genes in the core genome dataset. Multiple alignments of each orthologous gene set obtained from the core genome are then calculated using the MUSCLE software. The obtained alignments are then concatenated to one large complete core alignment which is then used to create a phylogenetic tree using the neighbor-joining method as implemented in the PHYLIP package.

When the whole genome sequence of strain H-02-3 was taxonomically analyzed using the EDGAR pipeline, it was found to taxonomically cluster with *Arthrobacter globiformis*, as shown by construction of a phylogenomic tree (Figure 3). *A*. *globiformis* has previously been shown to provide several critical ecosystem services to the environment. For example, it has the ability to oxidize ammonium into nitrite, nitrate and hydroylamine, which is critical for providing nitrogen source for plant growth. Furthermore, *A*. *globiformis* also has the ability to biodegrade pesticides and other contaminants found in soils, such as chromium and zinc (Asatiani et al., 2018). We are tempted to hypothesize that strain H-02-3 being close to *A*. *globiformis*, may also possess such environmentally beneficial traits. In fact, several other studies, including our ongoing work, provides unequivocal evidence to show critical ecosystem services being rendered by *Arthrobacter* spp. in soils, such as bioremediation of contaminants, cycling of heavy metals and even radionuclides (Hanbo et al., 2004; Swer et al., 2016; Chauhan et al., 2018). In this context, *Arthrobacter* spp., have been well-documented as key players in uraniferous environmental niches at another DOE site in Washington State, the Hanford site (Katsenovich et al., 2013); having a similar U contamination history as the SRS site. Similarly, *Arthrobacter* spp., also occur as cultivable members in other radionuclide contaminated environments across the globe (van Waasbergen et al., 2000; Fredrickson et al., 2004; Hanbo et al., 2004; Macur et al., 2004; Suzuki and Banfield, 2004; Swer et al., 2016). This environmental ubiquity of *Arthrobacter* strains is mostly related to the presence of plasmid(s) (Jerke et al., 2008), facilitating nutritional versatility, including biodegradation of contaminants (Chauhan and Jain, 2000; Chauhan et al., 2018), ability to resist prolonged periods of desiccation, starvation, and also ability to survive under a variety of environmental stressors (Boylen, 1973; Hagedorn and Holt, 1975; Cacciari and Lippi, 1987; Osman et al., 2008). Understanding the basis of *Arthrobacter*-mercury interactions at the genomic level, therefore, may facilitate identification of appropriate gene targets, which when manipulated, can potentially enhance bioremediative functions for the successful rehabilitation of Hg contaminated environments from the nuclear-legacy era, such as the SRS.

Further genomic analysis of strain H-02-3 using the RAST-based functional gene subsystem clustering revealed the presence of a total of 1732 subsystems represented by 41% of the strain’s genome. The top five subsystems belonged to carbohydrate metabolism (526); amino acids and derivatives (457); cofactors, vitamins, prosthetic groups, pigments (242); protein metabolism (237); and fatty acids, lipids, and isoprenoids (165). Additionally, functions related with membrane transport (119); stress response (89); resistance to antibiotics and toxic compounds (61); as well as metabolism of aromatic compounds (67) were also identified. Of particular interest to heavy metals, subsystems related to mercury resistance (MerB), the czc cobalt-zinc-cadmium resistance determinants and arsenic resistance were also identified in the genome of strain H-02-3. Collectively, these analyses of *Arthrobacter* sp. strain H-02-3, suggests the presence of several genome-enabled metabolic and catabolic processes, which may play a significant role in the colonization and its survival within the co-contaminated soil habitat, such as SRS.

### Potential Genes Providing Mercury Resistance in Strain H-02-3

**FIGURE 4:**
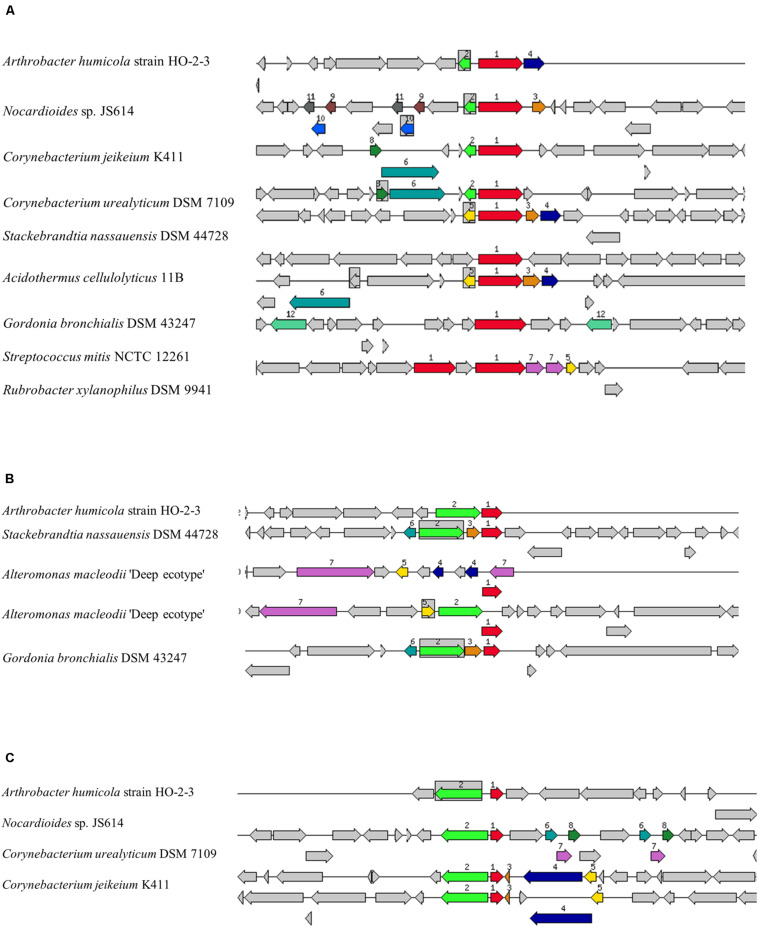
Mercury resistant gene determinants identified within the genome of *Arthrobacter* sp. strain H-02-3 relative to other bacteria. Shown are **(A)**, the mercuric ion reductase (EC 1.16.1.1) (*merA*). **(B)**, the organomercurial lyase (EC 4.99.1.2) (*merB*) and **(C)**, a mercuric resistance operon regulatory protein. The graphic is centered on the focus gene, which is red and numbered 1. Sets of genes with similar sequence are grouped with the same number and color. Genes whose relative position is conserved in at least eight other species are functionally coupled and share gray background boxes. The focus gene always points to the right, even if it is located on the minus strand.

The *Arthrobacter* genus are known for their ability to degrade xenobiotic compounds and resist several heavy-metals (Mongodin et al., 2006; Katsenovich et al., 2013). Several gene homologs were discovered by the genome-centric assessment of strain H-02-3, which have also been previously demonstrated for their role in resistance against radionuclides and heavy metal resistance, such as the efflux and MFS transporters, P-type ATPase translocators and heavy metal-responsive transcriptional regulators. The genome of strain H-02-3 harbored a 1,443 bp gene fragment on contig#28, with the assigned function of mercuric ion reductase (EC 1.16.1.1) (Figure 4A) MerA, along with the presence of 666 bp gene fragment, also present on contig#28, with the assigned function of an organomercurial lyase (EC 4.99.1.2), MerB (Figure 4B), which plays a vital role in HgR. A 390 bp gene fragment, also located on contig#28, was revealed during the whole genome sequencing data analysis of strain H-02-3, with the assigned function of HgR operon regulatory protein (Figure 4C). Furthermore, a gene fragment of 879 bp in size was also identified on contig#1, with the assigned function of transcriptional regulation. RAST annotation revealed this fragment to belong to the MerR family, which regulates *mer* by gene activation in the presence of Hg^2+^ and acts as a weak repressor in its absence (Lund and Brown, 1989).

BacMet, a repository of antibacterial biocide-resistance and metal-resistance genes was used to query the *merAB* genes against the predicted database, which revealed 35–37% homology of mercuric ion reductase (EC 1.16.1.1) and organomercurial lyase (EC 4.99.1.2) gene fragments of strain H-02-3 to genes in *Vibrio shilonii* AK1 (data not shown). Similarity of the *mer* operonic genes of the Gram-positive *Arthrobacter* strain H-02-3 with *Vibrio* sp., a Gram-negative bacterium are a bit surprising, however, this observation needs to be further validated experimentally.

**FIGURE 5:**
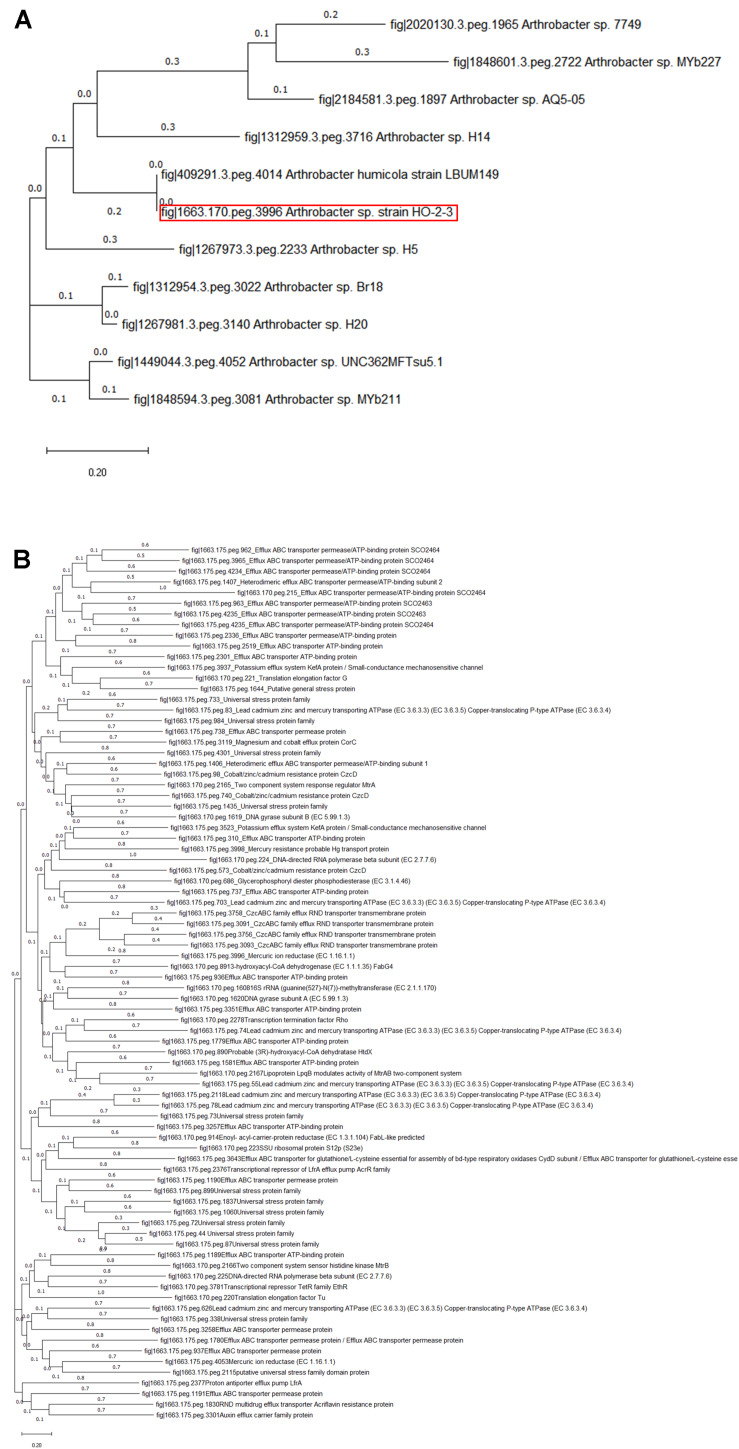
Shown are **(A)**, *merA* gene based phylogenetic tree created using the neighbor-joining method, which depicting the taxonomic relatives of strain H-02-3; **(B)**, phylotree of PATRIC annotated gene determinants in H-02-3 for functions related to mercury resistance, antimicrobial resistance, efflux, and universal stress response proteins, respectively. Tree was constructed using default parameters in MEGAX.

Because MerA has been demonstrated to perform a dominant role in narrow and broad-spectrum HgR in bacteria, gene homology of the MerA in strain H-02-3 was further evaluated using the PATRIC workflow; as shown in the phylogenetic tree in Figure 5A. This revealed that the *merA* gene in strain H-02-3 was closest to a similar gene present in *Arthrobacter humicola* strain LBUM149. Another notable feature of strain H-02-3 was the presence of several efflux pumps, arguably facilitating survival of this strain under the stress of mixed contaminants, by shunting out contaminants from the intracellular environment; a phylogenetic tree representing gene determinants for efflux pumps, antimicrobials, universal stress proteins and Hg was constructed and shown in Figure 5B, which is discussed in further details later in this writing.

### Genomic Islands (GEIs) in *Arthrobacter* sp. Strain H-02-3

**FIGURE 6:**
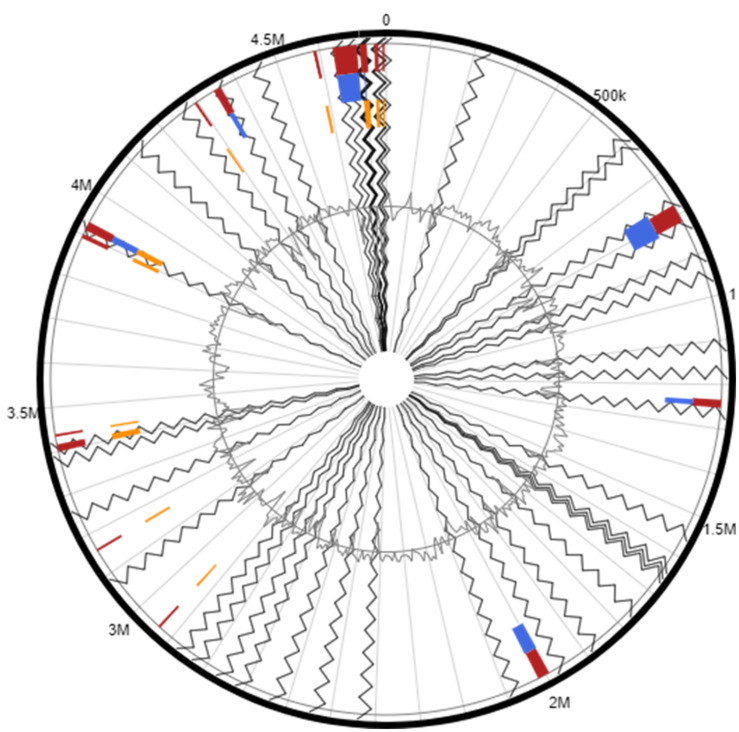
Shown are the putative genomic islands (GEIs) harbored by *Arthrobacter* sp. strain H-02-3; *A*. *aurescens* TC1 was used as the reference genome to run this comparative analysis. The outer black circle represents the scale line in Mbps and GEIs obtained from each of the following methods are shown in color: SIGI-HMM (orange), IslandPath-DIMOB (blue), and integrated detection (red), respectively.

Another interesting genomic trait, the presence of several GEIs was identified in the strain H-02-3. Unlike any genome, bacterial genomes consist of a set of core genes which encode for essential metabolic functions. In bacteria, these genes are supported by additional genes acquired via horizontal gene transfer (HGT) mechanisms. These supplementary genes may render additional metabolic functions such as adaptive traits and genomic plasticity, which may facilitate evolutionary survival (Pérez-Pantoja et al., 2013). Generally, GEIs have been associated with traits such as virulence or antibiotic resistance rendered to the host bacteria. However, recent whole genome sequencing studies have revealed other GEI-encoded functional traits, which can be classified into four major categories; pathogenicity islands (PAIs), coding for virulence genes; metabolic islands (MIs), consisting of genes for biosynthesis of secondary metabolites; resistance islands (RIs), coding for resistance genes usually toantibiotics; and symbiotic islands (SIs), genes coding for symbiotic associations of the host with other micro and/or macroorganisms. In the current study we were able to identify several GEIs in the strain H-02-3 when compared against the complete genome of the reference strain *A*. *aurescens* TC1 (Figure 6). Several of the identified GEI homologs in strain H-02-3 further analyzed by BLAST, were found to be closely affiliated with genes that have been previously implicated in metal-resistance as well as biodegradation of contaminant hydrocarbons. These results suggest a strong possibility that GEIs were likely recruited via HGT, so as to facilitate survival in extreme environments, such as the SRS studied, which is co-contaminated by heavy metals and organic compounds.

**FIGURE 7:**
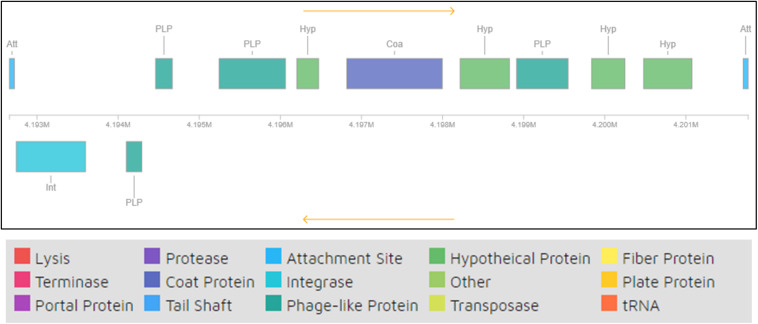
Remnants of bacteriophage regions identified from querying the whole genome sequence of *Arthrobacter* sp. strain H-02-3. The boxes are color coded with the legend pasted below the figure to show their potential functions.

One of the mechanisms for GEIs to be present in bacterial genomes is via bacteriophage mediated transfer. It has been shown that bacteriophages contribute toward bacterial evolutionary mechanisms by engaging in HGT, mainly bringing about external genetic material that encodes new characteristics to their hosts, which are many times beneficial and thus retained by the host. Some such traits include virulence factors, antibiotic resistance, and GEIs (Bernheim and Sorek, 2018). Analysis of phage remnants in strain H-02-3 revealed the presence of a prophage region with 12 CDS, as shown in Figure 7. Thus, it appears that strain H-02-3 recruited phage mediated genetic elements followed by genetic reshuffling that incorporated these “foreign” genes which likely contribute toward successful adaptation and survival of H-02-3 in a highly contaminated soil environment from where it was isolated.

### Genomic Comparison of *Arthrobacter* sp. Strain H-02-3

**FIGURE 8:**
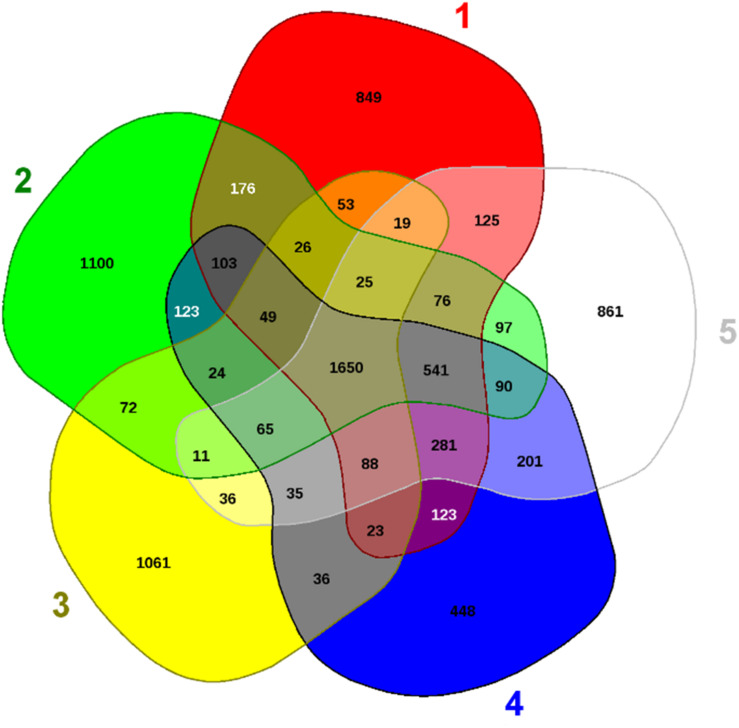
Shown are the whole genome sequence-based Venn diagram generated between *Arthrobacter* sp. H-02-3 with its closest functional relatives. Venn diagram sectors belong to (1) *Arthrobacter globiformis* strain NBRC-12137; (2) *Arthrobacter* sp. strain H-02-3; (3) *Arthrobacter woluwensis* strain DSM; (4) *Pseudoarthrobacter phenanthrenivorans* strain SWC37, and (5) *Pseudarthrobacter siccitolerans* strain 4J27. The number of singleton genes appear in red, green, yellow, blue and white areas for strains 1–5 listed above -along with their core genomes (centered gray area).

To further infer the evolutionary relatedness of strain H-02-3 relative to closely related phylogenetic species, we ran EDGAR analysis and results are shown in Figure 8. The strain H-02-3 was shown to harbor 1100 distinct genes which were not found in the other four closely associated *Arthrobacter* species. Moreover, several distinctive genes were also identified in the other four *Arthrobacter* species, shown in parenthesis: *Pseudarthrobacter siccitolerans* 4J27 (861); *Arthrobacter woluwensis* strain DSM 10495 (1062); *A*. *globiformis* strain NBRC 1237 (849), and *Pseudoarthrobacter phenanthrenivorans* strain SWC37 (448), respectively.

The 1100 distinct genes present in H-02-3 (#2 sector in Figure 8), identified from the EDGAR analysis, made up approximately 26.7% of the total genome size of the strain. Notably, majority of these “distinct” gene determinants were determined to encode for the metal resistance proteins (for metals like cadmium, cobalt, and zinc), stress proteins, transporter proteins, cytochromes, and for drug resistance (Table 1). The comparative genomic analysis of the strain H-02-3 with other four strains, confirms a strong catabolic and bioremediation potential possessed by the isolated strain.

**TABLE 1:** Shown are selected gene homologs that likely perform biodegradative or metal resistance function in *Arthrobacter* sp. strain H-02-3.

**Category**	**Gene homolog**	**Gene ID**
Transporter proteins	Putative nitrate/nitrite transporter NarK2	PKKPIOGD_00674
	Putative transporter YycB	PKKPIOGD_01646
	ABC transporter periplasmic-binding protein YphF	PKKPIOGD_02404
	Putative transporter	PKKPIOGD_02845
	Putative ABC transporter permease	PKKPIOGD_03198
	Putative ABC transporter ATP-binding protein YbhF	PKKPIOGD_03290
Stress proteins	Universal stress protein	PKKPIOGD_00044
	General stress protein 14	PKKPIOGD_00992
	General stress protein 69	PKKPIOGD_02454
Cytochromes	Cytochrome P450 116	PKKPIOGD_00508
	Cytochrome b6-f complex iron-sulfur subunit	PKKPIOGD_00599
	Bifunctional cytochrome P450/NADPH–P450 reductase 2	PKKPIOGD_00757
	Cytochrome p450 CYP199A2	PKKPIOGD_01088
	Cytochrome b6	PKKPIOGD_01331
	Cytochrome P450 107B1	PKKPIOGD_03276
Metal resistance proteins	Multicopper oxidase mco	PKKPIOGD_00039
	Multicopper oxidase MmcO	PKKPIOGD_00059
	Copper-sensing transcriptional repressor RicR	PKKPIOGD_00071
	Copper chaperone CopZ	PKKPIOGD_00072
	Copper resistance protein C	PKKPIOGD_00095
	Nickel-binding periplasmic protein	PKKPIOGD_03385
	Cobalt-zinc-cadmium resistance protein CzcA	PKKPIOGD_03036
	Cobalt/magnesium transport protein CorA	PKKPIOGD_01683
	Arsenical pump-driving ATPase	PKKPIOGD_00505
	Arsenical resistance operon trans-acting repressor ArsD	PKKPIOGD_00506
	Arsenical pump membrane protein	PKKPIOGD_01058
Drug resistance	Multidrug resistance protein Stp	PKKPIOGD_01096
	Multidrug resistance protein MdtL	PKKPIOGD_04227

*The analysis was conducted using default mode of EDGAR and the suite of distinct genes in strain H-02-3 were manually queried for desired bioremediation functions listed under the category heading. The genome-to-genome comparison analysis presented in this table was conducted on strain H-02-3 in reference to *Arthrobacter globiformis* strain NBRC-12137; *Arthrobacter* sp. strain H-02-3; *Arthrobacter woluwensis* strain DSM; *Pseudoarthrobacter phenanthrenivorans* strain SWC37 and *Pseudarthrobacter siccitolerans* strain 4J27, respectively.*

### Metal Pollutome and Antibiotic Resistome Determinants in *Arthrobacter* sp. Strain H-02-3

The genomic analysis of strain H-02-3 resulted in the identification of a suite of metal and antibiotic resistant genes. It has also been established that gene determinants that render resistance to Hg are almost always concomitantly present with ARGs, specifically against ampicillin, tetracycline, erythromycin, and penicillin (Ready et al., 2003). Therefore, presence of Hg and U stressors present in the SRS soils pose public health and ecological concerns from the pollutome driven antibiotic resistomes or vice versa. The detailed genomic analysis of *Arthrobacter* sp. strain H-02-3 revealed many gene determinants of transporters, virulence factors, drug targets, and antibiotic resistance. A summary of the classes of ARGs annotated from strain H-02-3 and the corresponding five mechanisms are provided in Table 2.

**TABLE 2:** Shown are the PATRIC-annotated antimicrobial resistant (AMR) gene determinants and their associated mechanisms identified from *Arthrobacter* sp. strain H-02-3.

**AMR mechanism**	**Genes**
Antibiotic target in susceptible species	Alr, Ddl, dxr, EF-G, EF-Tu, folA, Dfr, folP, gyrA, gyrB, Iso-tRNA, kasA, MurA, rho, rpoB, rpoC, S10p, S12p
Antibiotic target replacement protein	FabG, FabL-like, HtdX
Gene conferring resistance via absence	gidB
Protein altering cell wall charge conferring antibiotic resistance	GdpD, PgsA
Regulator modulating expression of antibiotic resistance genes	EthR, LpqB, MtrA, MtrB

Furthermore, one major mechanism underpinning microbial resistance against both heavy metals and antibiotics is by effluxing out these compounds once encountered by the cellular environment (Levy, 2002; Nies, 2003; Blanco et al., 2016; Buffet-Bataillon et al., 2016). At the genome level, strain H-02-3 possessed an arsenal of efflux pumps, which were manually curated and phlogeny was studied, as shown in Figure 5B. This also revealed that the mercuric ion reductase (*merA*) gene in strain H-02-3 (fig | 1663.175.peg.4053) was homologous to a putative universal stress family domain protein (fig | 1663.175.peg.2115) as well as efflux ABC transporter permease protein (fig | 1663.175.peg.937). This is suggestive of evolutionary and functional similarities between the key mercury resistance gene, *merA* with the above stated efflux and stress proteins in strain H-02-3, which likely collectively aids the strain to survive in a metal contaminated soil habitat.

### Statistical Analysis of Selected Gene Classes in *Arthrobacter* sp. Strain H-02-3

**FIGURE 9:**
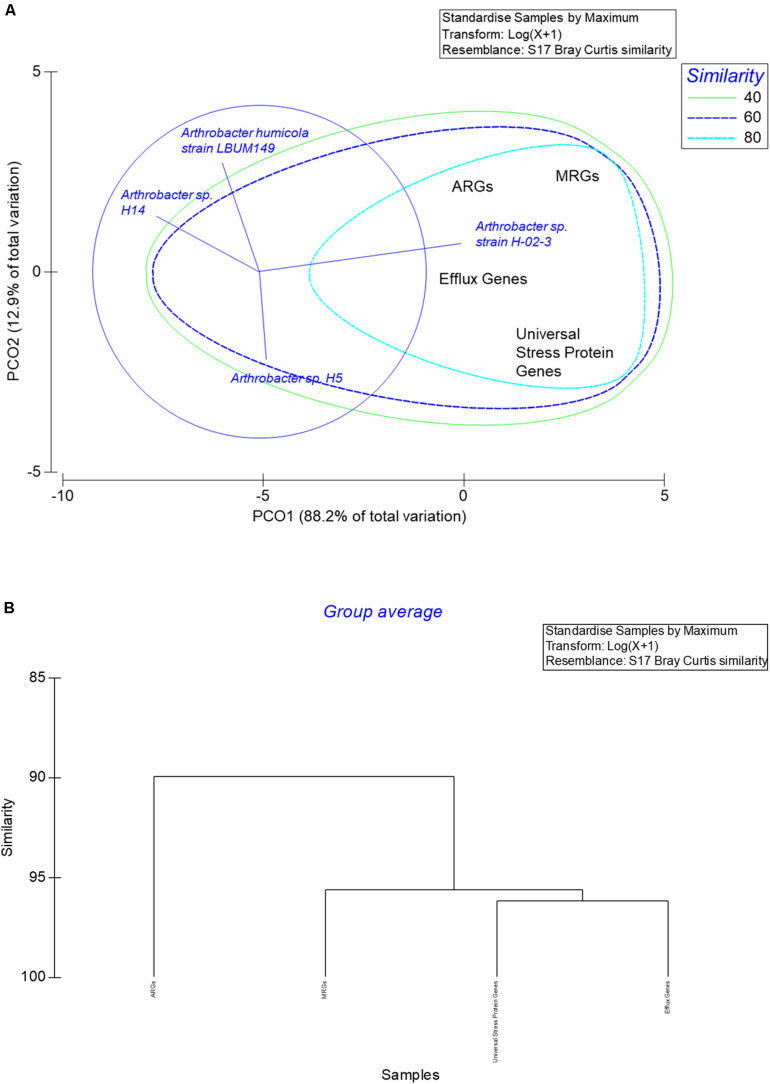
Shown are **(A)**, the PCoA ordination plot of the number of genes related to mercury resistance, antimicrobial resistance, efflux, and universal stress response proteins in strain H-02-3 relative to three closest phylogenetic relatives. Above stated gene were binned based on the *merA* gene homology in strain H-02-3 and analyzed using the Bray Curtis similarity matrix. **(B)**, cluster analysis of the selected gene classes in H-02-3 relative to three closest phylogenetic relatives.

The number of PATRIC annotated gene classes for metal resistance (including mercury), AMR, efflux, and universal stress response proteins in strain H-02-3 were collated and analyzed relative to three closest phylogenetic relatives. The relatives were chosen based on the *merA* gene homology, confirmed by PCoA, which revealed that all selected genes of environmental relevance in strain H-02-3, especially the ARGs and MRGs gene sequences, clustered together on the same axis (Figure 9A). Conversely, the other three taxonomic relatives clustered away from H-02-3, indicating the unique genomic nature of the SRS native strain. Further, the vectors clearly indicated the number of genes driving the separation between the strains evaluated. The close correlation between the ARGs and MRGs were further seen in the cluster analysis, as depicted in Figure 9B. This is clear depiction of tighter association of studied genes, potentially rendering genomic traits for soil survival for strain H-02-3; a trend not closely mirrored by the phylogenetic relatives selected for this study. The rationale for strain H-02-3 to possess a strong association between MRGs and ARGs points to these genes being co-selected in the SRS habitat that are stressed by the presence of metal co-contaminants such as Hg and U, respectively. Thus, such HgR strains can be used as models to understand the impacts of co-contaminants to the evolutionary recruitment of ARGs and MRGs within the SRS system. However, at this stage, it is hard to accurately tease out the drivers that determine the co-selection of ARGs and MRGs within the resident microbial communities in the SRS ecosystem, which requires further studies. As a cautionary note, it is emphasized that this study was not performed to delineate whether ARGs and MRGs are located on the same genetic element i.e., chromosomally located, plasmid(s)-borne, or as genomic island(s), or if they are physically distanced within the strain’s genome or that the cross resistance of ARGs and MRGs are controlled by a single or distinct regulatory element(s); these aspects remain unclear at this time. Furthermore, how do metal and antibiotic resistances interact, given that the strain is resident in a soil environment that is co-contaminated with a variety of heavy metals and other pollutants, also remains unclear from this work. These seminal questions are central to obtain a better understanding on the nexus between heavy metals and antibiotic resistance which forms the basis for future studies, which are ongoing.

Overall, we propose that the identified gene determinants in *Arthrobacter* sp. H-02-3 provide ecologically and environmentally critical traits for cellular survival in contaminant-rich SRS soils. Furthermore, it has been demonstrated that metal stress may also play a role in enhancing the resistome of the bacteria i.e., the arsenal of ARGs (Hu et al., 2017). This heavy metal-driven co-selection may lead to acceleration of the evolutionary processes, which may lead to the development of highly potent multi-drug resistant “superbugs,” in the near future, with pathogenic genomic attributes to cause human and animal diseases. Therefore, it can be concluded that heavy metal co-contaminants that are typically present in DOE managed ecosystems such as the SRS, not only pose ecological and public health toxicity risks but also stress the environmental microbiomes, which respond by acquiring MRGs via HGT or genomic mutations for survival. The MRGs can possess antimicrobial activities or facilitate recruitment of additional antimicrobial resistant genes (ARGs), thus exacerbating the environmental toxicity issues. Therefore, this study recommends that collective mitigation strategies against both heavy metals, such as Hg, as well as antibiotics should be implemented for better stewardship of historically contaminated environments.

## Data Availability Statement

The datasets generated for this study can be found in the draft genome sequence of *Arthrobacter* sp. strain H-02-3 reported in this study has been deposited at DDBJ/ENA/GenBank under the accession PYUI00000000 (https://www.ncbi.nlm.nih.gov/nuccore/PYUI00000000); Bio Project: PRJNA445730 (https://www.ncbi.nlm.nih.gov/bioproject/PRJNA445730); BioSample: SAMN08797595 (https://www.ncbi. nlm.nih.gov/biosample/8797595). This WGS version of the project consists of sequences PYUI01000001–PYUI01000044.

## Author Contributions

AC, AP, and RJ designed the experiments and drafted the manuscript. AP and AC performed the genomics, comparative genomics, and statistical analysis. RJ isolated the strain, performed the physiological tests, and analyzed the results.

## Conflict of Interest

The authors declare that the research was conducted in the absence of any commercial or financial relationships that could be construed as a potential conflict of interest.

## Footnotes

1 http://www.ncbi.nlm.nih.gov/

2 http://www.pathogenomics.sfu.ca/islandviewer/
