# Corrigendum: Genomic Characterization of a Mercury Resistant *Arthrobacter* sp. H-02-3 Reveals the Presence of Heavy Metal and Antibiotic Resistance Determinants

**DOI:** 10.3389/fmicb.2020.01047

**Published:** 2020-05-25

**Authors:** Ashish Pathak, Rajneesh Jaswal, Ashvini Chauhan

**Affiliations:** Environmental Biotechnology Laboratory, School of the Environment, FSH Science Research Center, Florida A&M University, Tallahassee, FL, United States

**Keywords:** mercury, whole genome sequencing, comparative genomics, *Arthrobacter*, resistome

In the original article, there was an error in the title. A correction has been made to title:

“Genomic Characterization of a Mercury Resistant *Arthrobacter* sp. H-02-3 Reveals the Presence of Heavy Metal and Antibiotic Resistance Determinants”.

The authors apologize for this error and state that this does not change the scientific conclusions of the article in any way. The original article has been updated.

